# Bis{*N*-[methoxy(4-methylbenzamido)methyl]-2,4-dimethylanilinido-κ^2^ *N*,*O*}copper(II)

**DOI:** 10.1107/S1600536812025081

**Published:** 2012-06-13

**Authors:** M. Sukeri M. Yusof, Maisara A. Kadir, Bohari M. Yamin

**Affiliations:** a Department of Chemical Sciences, Faculty of Science and Technology, Universiti Malaysia Terengganu, Mengabang Telipot, 21030 Kuala Terengganu, Terengganu, Malaysia; b School of Chemical Sciences and Food Technology, Universiti Kebangsaan Malaysia, UKM 43600 Bangi Selangor, Malaysia

## Abstract

In the centrosymmetric mononuclear title complex, [Cu(C_18_H_20_N_2_O_2_)_2_], the Cu^II^ atom is four-coordinated in a *trans-*CuN_2_O_2_ square-planar geometry with the N—Cu—O chelate angle being 89.97 (11)°. The dihedral angles made by the planes defined by the aromatic ring carbons of the 4-methyl­benzene and 2,4-dimethyl­benzene fragments with the plane defined by the chelate ring are 13.43 (15) and 82.69 (13)° respectively. The angle between the planes defined by the aromatic carbons of the two rings is 89.40 (16)°. A a weak intra­molecular C—H⋯N hydrogen bond occurs.

## Related literature

For applications of related compounds, see: Moro *et al.* (2009 ▶); Rauf *et al.* (2009 ▶); D’Cruz *et al.* (2003 ▶). For a related structure, see: Shen *et al.* (1999 ▶). For C—N bond lengths, see: Arslan *et al.* (2007 ▶).
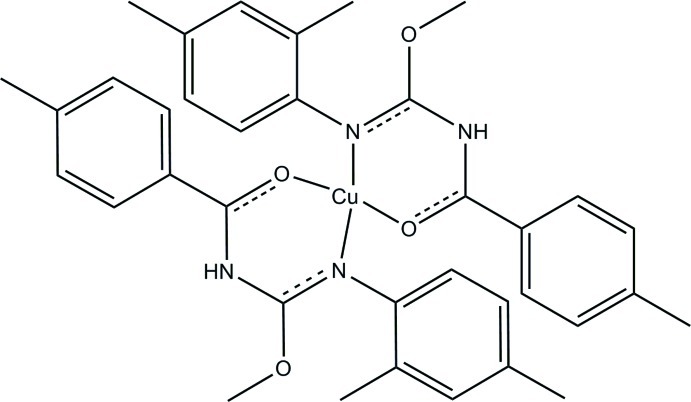


## Experimental

### 

#### Crystal data

[Cu(C_18_H_20_N_2_O_2_)_2_]
*M* *_r_* = 656.26
Triclinic, 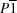

*a* = 7.949 (2) Å
*b* = 10.191 (3) Å
*c* = 10.844 (4) Å
α = 80.784 (6)°
β = 74.302 (6)°
γ = 79.772 (6)°
*V* = 826.4 (4) Å^3^
*Z* = 1
Mo *K*α radiation
μ = 0.71 mm^−1^
*T* = 298 K
0.40 × 0.24 × 0.18 mm

#### Data collection

Bruker SMART APEX CCD area-detector diffractometer
Absorption correction: multi-scan (*SADABS*; Bruker, 2000 ▶) *T*_min_ = 0.766, *T*_max_ = 0.884
10650 measured reflections
3792 independent reflections
2609 reflections with *I* > 2/s(*I*)
*R*_int_ = 0.029

#### Refinement

*R*[*F* ^2^ > 2σ(*F* ^2^)] = 0.063
*wR*(*F* ^2^) = 0.198
*S* = 1.01
3792 reflections
205 parameters
H-atom parameters constrained
Δρ_max_ = 1.10 e Å^−3^
Δρ_min_ = −0.43 e Å^−3^

### 

Data collection: *SMART* (Bruker, 2000 ▶); cell refinement: *SAINT* (Bruker, 2000 ▶); data reduction: *SAINT*; program(s) used to solve structure: *SHELXS97* (Sheldrick, 2008 ▶); program(s) used to refine structure: *SHELXL97* (Sheldrick, 2008 ▶); molecular graphics: *SHELXTL* (Sheldrick, 2008 ▶); software used to prepare material for publication: *SHELXTL*, *PARST* (Nardelli, 1995 ▶) and *PLATON* (Spek, 2009 ▶).

## Supplementary Material

Crystal structure: contains datablock(s) global, I, 265R. DOI: 10.1107/S1600536812025081/qm2070sup1.cif

Structure factors: contains datablock(s) I. DOI: 10.1107/S1600536812025081/qm2070Isup2.hkl

Additional supplementary materials:  crystallographic information; 3D view; checkCIF report

## Figures and Tables

**Table 1:** Hydrogen-bond geometry (Å, °)

*D*—H⋯*A*	*D*—H	H⋯*A*	*D*⋯*A*	*D*—H⋯*A*
C18—H18*A*⋯N1	0.96	2.27	2.783 (7)	112

**Fig. 1.**
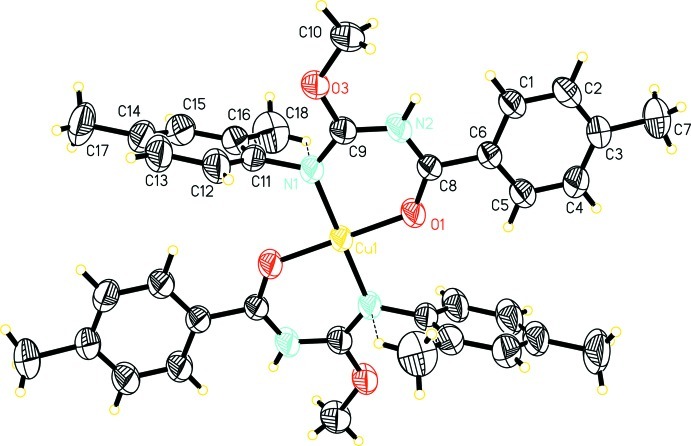
The molecular structure of (I) with the atom labeling scheme. Displacement ellipsods are drawn at the 50% probability level. Hydrogen bonds are shown as dashed lines.

## supplementary crystallographic information

### Comment

The interest in the synthesis and properties of transition metal complexes containing thiourea derivatives has received a significant level of attention. This is due to their significant biological activity and variation in the mode of binding. Thiourea derivatives have shown great potential in medicinal applications especially as anticancer, antifungal, antibacterial and most interestingly as anti-HIV agents (Moro *et al.*, 2009; Rauf *et al.*, 2009; D'Cruz *et al.*, 2003). In this work, the title compound derived from the desulfurization of *N*-(4-methylbenzoyl)-*N'*-(2,4-dimethylphenyl)thiourea has been successfully synthesised. The molecular structure shows that the ligand acts as a bidentate chelating ligand through nitrogen and oxygen atoms (Fig. 1). The molecule is discrete and centrosymmeric about the Cu1 atom. The two ligands coordinate to the metal centre with the N1-Cu1-O1 bond angles of 89.97 (11)°. This is typical square planar geometry for a four coordinate complex. The Cu1-N1 and Cu1-O1 bond lengths are 1.963 (2) and 1.892 (2)Å, respectively, which is in agreement with a related complex (Shen *et al.*, 1999). The bond length O1-C8 is 1.263 (3)Å, which is slightly shorter than the other bonds. This indicates the presence of partial double bond character in the this bond due to resonance effects. The C-N bond lengths of the [C9-N2 = 1.310 (4)Å, C9-N1= 1.325 (4)Å and C8-N1 = 1.313 (4)Å] groups lie in the range expected for C-N bonds with partial double bond character. This is shorter than the bond lengths for normal C-N bonds (about 1.48Å) reported in the literature (Arslan *et al.*, 2007). The presence of strong delocalization in the chelate ring N2, C9, N1, C8 and O1 atoms, suggested the presence of a conjugated π-system along N2-C9-N1-C8-O1 which is similar to other reported carbonylthiourea derivatives (Shen *et al.*, 1999).

The six-membered ring Cu1-N2-C9-N1-C8-O1-Cu1, 4-methylphenyl (C1-C7), and 2,4-dimethylphenyl (C10-C15/C16/C17) groups are essentially planar with maximum deviation of 0.016 (2)Å for atom N2 from the least-squares planes. The chelating ring makes dihedral angles with the 4-methylphenyl and 2,4-dimethylphenyl fragments of 13.43 (15) and 82.69 (13)°, respectively. The plane of the 4-methyphenyl and 2,4-dimethyphenyl fragments are inclined to each other at an angle of 89.40 (16)°. There is a weak intramolecular hydrogen bond C18-H18A···N1 resulting a pseudo five-membered ring, C11-C16-C18-H18A···N1 (Table 1).

### Experimental

A solution of *N*-(4-methylbenzoyl)-*N'*-(2,4-dimethylphenyl)thiourea (0.51 g, 2 mmol) in DCM (30 ml) was added dropwise to a solution of copper(II) acetate monohydrate (0.17 g, 1 mmol) in MeOH (50 ml) at about 15 minutes rate in 100 ml two-necked round bottom flask with constant stirring at room temperature. The reaction was slowly put under reflux for ca. 3 hours. Reaction progress was monitored by TLC (hexane: ethyl acetate; 7:3). When the reaction had completed, the solvent was removed in-vacuo and followed by recrystallisation from DCM: MeOH (1:3) to afford dark green solid crystals. The crude product was purified by preparative TLC (hexane: acetone; 7:3) and the dark green band was collected and recrystallised from MeOH to afford the title compound (0.16 g, 24°).

### Refinement

All H atoms attached to C and N atoms were fixed geometrically and treated as riding with C-H= 0.93-0.97 Å(aromatic and methylene) and N—H= 0.86 Å(amino) with *U*_iso_(H)=1.2*U*_eq_(C or N). There are highest peak 1.26Å and deepest hole 0.93Å for Br1 atom.

### Crystal data

**Table:**

[Cu(C_18_H_20_N_2_O_2_)_2_]	*V* = 826.4 (4) Å^3^
*M**_r_* = 656.26	*Z* = 1
Triclinic, *P*1	*F*(000) = 345
Hall symbol: -P 1	*D*_x_ = 1.319 Mg m^−^^3^
*a* = 7.949 (2) Å	Mo *K*α radiation, λ = 0.71073 Å
*b* = 10.191 (3) Å	θ = 2.0–27.5°
*c* = 10.844 (4) Å	µ = 0.71 mm^−^^1^
α = 80.784 (6)°	*T* = 298 K
β = 74.302 (6)°	Slab, dark green
γ = 79.772 (6)°	0.40 × 0.24 × 0.18 mm

### Data collection

**Table:**

Bruker SMART APEX CCD area-detector diffractometer	3792 independent reflections
Radiation source: fine-focus sealed tube	2609 reflections with *I* > 2/s(*I*)
Graphite monochromator	*R*_int_ = 0.029
Detector resolution: 83.66 pixels mm^-1^	θ_max_ = 27.5°, θ_min_ = 2.0°
ω scan	*h* = −10→10
Absorption correction: multi-scan (*SADABS*; Bruker, 2000)	*k* = −13→13
*T*_min_ = 0.766, *T*_max_ = 0.884	*l* = −14→14
10650 measured reflections	

### Refinement

**Table:**

Refinement on *F*^2^	Primary atom site location: structure-invariant direct methods
Least-squares matrix: full	Secondary atom site location: difference Fourier map
*R*[*F*^2^ > 2σ(*F*^2^)] = 0.063	Hydrogen site location: inferred from neighbouring sites
*wR*(*F*^2^) = 0.198	H-atom parameters constrained
*S* = 1.01	*w* = 1/[σ^2^(*F*_o_^2^) + (0.1258*P*)^2^ + 0.1796*P*] where *P* = (*F*_o_^2^ + 2*F*_c_^2^)/3
3792 reflections	(Δ/σ)_max_ < 0.001
205 parameters	Δρ_max_ = 1.10 e Å^−^^3^
0 restraints	Δρ_min_ = −0.43 e Å^−^^3^

### Special details

**Table:**

Geometry. All esds (except the esd in the dihedral angle between two l.s. planes) are estimated using the full covariance matrix. The cell esds are taken into account individually in the estimation of esds in distances, angles and torsion angles; correlations between esds in cell parameters are only used when they are defined by crystal symmetry. An approximate (isotropic) treatment of cell esds is used for estimating esds involving l.s. planes.
Refinement. Refinement of F^2^ against ALL reflections. The weighted R-factor wR and goodness of fit S are based on F^2^, conventional R-factors R are based on F, with F set to zero for negative F^2^. The threshold expression of F^2^ > 2sigma(F^2^) is used only for calculating R-factors(gt) etc. and is not relevant to the choice of reflections for refinement. R-factors based on F^2^ are statistically about twice as large as those based on F, and R- factors based on ALL data will be even larger.

### Fractional atomic coordinates and isotropic or equivalent isotropic displacement parameters (Å^2^)

**Table:**

	*x*	*y*	*z*	*U*_iso_*/*U*_eq_	
Cu1	0.0000	0.5000	0.0000	0.0627 (3)	
O1	−0.1657 (4)	0.3971 (2)	0.1152 (3)	0.0901 (10)	
N2	−0.0057 (4)	0.1849 (3)	0.1172 (3)	0.0681 (8)	
H2B	−0.0059	0.1033	0.1531	0.082*	
N1	0.1674 (4)	0.3356 (3)	−0.0332 (3)	0.0656 (8)	
C1	−0.3153 (5)	0.0812 (4)	0.2570 (4)	0.0821 (13)	
H1A	−0.2125	0.0215	0.2332	0.098*	
C2	−0.4700 (6)	0.0327 (4)	0.3219 (5)	0.1020 (17)	
H2A	−0.4695	−0.0595	0.3429	0.122*	
C3	−0.6273 (5)	0.1185 (4)	0.3570 (4)	0.0756 (11)	
C4	−0.6217 (5)	0.2527 (4)	0.3283 (4)	0.0815 (12)	
H4A	−0.7240	0.3124	0.3535	0.098*	
C5	−0.4678 (5)	0.3015 (4)	0.2629 (4)	0.0791 (12)	
H5A	−0.4689	0.3938	0.2423	0.095*	
C6	−0.3110 (4)	0.2167 (3)	0.2269 (3)	0.0592 (8)	
C7	−0.7988 (6)	0.0625 (6)	0.4239 (6)	0.1115 (19)	
H7A	−0.8935	0.1353	0.4412	0.167*	
H7B	−0.7873	0.0096	0.5035	0.167*	
H7C	−0.8239	0.0073	0.3690	0.167*	
C8	−0.1487 (4)	0.2717 (3)	0.1485 (3)	0.0589 (9)	
C9	0.1395 (4)	0.2185 (3)	0.0320 (4)	0.0630 (9)	
O3	0.2725 (3)	0.1187 (2)	0.0036 (3)	0.0813 (9)	
C10	0.2530 (6)	−0.0141 (4)	0.0702 (5)	0.0887 (14)	
H10A	0.3583	−0.0746	0.0400	0.133*	
H10B	0.1542	−0.0441	0.0537	0.133*	
H10C	0.2335	−0.0120	0.1612	0.133*	
C11	0.3306 (4)	0.3394 (3)	−0.1320 (4)	0.0662 (10)	
C12	0.4865 (5)	0.3460 (4)	−0.1022 (5)	0.0841 (13)	
H12A	0.4905	0.3481	−0.0176	0.101*	
C13	0.6398 (5)	0.3496 (4)	−0.2046 (6)	0.0915 (15)	
H13A	0.7466	0.3515	−0.1856	0.110*	
C14	0.6383 (6)	0.3502 (4)	−0.3302 (6)	0.0976 (17)	
C15	0.4796 (6)	0.3439 (4)	−0.3572 (5)	0.0901 (14)	
H15A	0.4755	0.3433	−0.4420	0.108*	
C16	0.3248 (5)	0.3384 (4)	−0.2577 (5)	0.0763 (12)	
C17	0.8026 (7)	0.3562 (6)	−0.4423 (6)	0.129 (2)	
H17A	0.9024	0.3606	−0.4100	0.193*	
H17B	0.7844	0.4346	−0.5021	0.193*	
H17C	0.8241	0.2774	−0.4854	0.193*	
C18	0.1646 (7)	0.3292 (7)	−0.2882 (6)	0.1151 (18)	
H18A	0.0711	0.3267	−0.2106	0.173*	
H18B	0.1778	0.2489	−0.3276	0.173*	
H18C	0.1366	0.4060	−0.3469	0.173*	

### Atomic displacement parameters (Å^2^)

**Table:**

	*U*^11^	*U*^22^	*U*^33^	*U*^12^	*U*^13^	*U*^23^
Cu1	0.0487 (4)	0.0292 (3)	0.0850 (5)	−0.0033 (2)	0.0177 (3)	0.0044 (2)
O1	0.0619 (15)	0.0325 (11)	0.133 (2)	−0.0030 (10)	0.0375 (15)	0.0051 (13)
N2	0.0540 (16)	0.0318 (13)	0.093 (2)	−0.0040 (11)	0.0103 (14)	0.0142 (13)
N1	0.0492 (15)	0.0347 (13)	0.087 (2)	−0.0020 (11)	0.0173 (13)	0.0042 (12)
C1	0.067 (2)	0.0387 (17)	0.110 (3)	−0.0080 (16)	0.022 (2)	0.0062 (18)
C2	0.083 (3)	0.045 (2)	0.142 (4)	−0.021 (2)	0.034 (3)	−0.001 (2)
C3	0.067 (2)	0.064 (2)	0.079 (3)	−0.0243 (18)	0.0207 (18)	−0.0087 (19)
C4	0.061 (2)	0.060 (2)	0.096 (3)	−0.0054 (17)	0.0249 (19)	−0.008 (2)
C5	0.067 (2)	0.0414 (18)	0.102 (3)	−0.0108 (16)	0.022 (2)	−0.0004 (18)
C6	0.0580 (19)	0.0388 (16)	0.064 (2)	−0.0093 (13)	0.0105 (15)	0.0010 (14)
C7	0.076 (3)	0.104 (4)	0.132 (4)	−0.044 (3)	0.034 (3)	−0.018 (3)
C8	0.0538 (18)	0.0326 (14)	0.072 (2)	−0.0066 (13)	0.0112 (15)	0.0001 (13)
C9	0.0490 (17)	0.0349 (15)	0.086 (2)	−0.0020 (13)	0.0062 (16)	0.0032 (15)
O3	0.0555 (14)	0.0312 (11)	0.123 (2)	0.0030 (10)	0.0185 (14)	0.0111 (12)
C10	0.075 (3)	0.0341 (17)	0.125 (4)	0.0045 (16)	0.007 (2)	0.0141 (19)
C11	0.0522 (19)	0.0314 (14)	0.087 (3)	−0.0001 (13)	0.0195 (16)	0.0035 (15)
C12	0.048 (2)	0.048 (2)	0.128 (4)	−0.0017 (15)	0.008 (2)	0.014 (2)
C13	0.049 (2)	0.057 (2)	0.136 (4)	−0.0009 (17)	0.012 (2)	0.014 (2)
C14	0.066 (3)	0.048 (2)	0.134 (4)	0.0010 (18)	0.036 (3)	0.005 (2)
C15	0.086 (3)	0.058 (2)	0.098 (3)	−0.014 (2)	0.024 (2)	−0.004 (2)
C16	0.057 (2)	0.049 (2)	0.106 (3)	−0.0094 (16)	0.011 (2)	−0.0074 (19)
C17	0.074 (3)	0.103 (4)	0.154 (5)	−0.006 (3)	0.053 (3)	0.001 (3)
C18	0.100 (4)	0.120 (5)	0.123 (4)	−0.028 (4)	−0.015 (3)	−0.014 (4)

### Geometric parameters (Å, º)

**Table:**

Cu1—O1	1.891 (2)	C7—H7C	0.9600
Cu1—O1^i^	1.891 (2)	C9—O3	1.338 (4)
Cu1—N1^i^	1.962 (3)	O3—C10	1.442 (4)
Cu1—N1	1.962 (3)	C10—H10A	0.9600
O1—C8	1.264 (4)	C10—H10B	0.9600
N2—C8	1.312 (4)	C10—H10C	0.9600
N2—C9	1.328 (4)	C11—C12	1.377 (6)
N2—H2B	0.8600	C11—C16	1.378 (6)
N1—C9	1.309 (4)	C12—C13	1.411 (6)
N1—C11	1.444 (4)	C12—H12A	0.9300
C1—C6	1.371 (5)	C13—C14	1.364 (8)
C1—C2	1.374 (5)	C13—H13A	0.9300
C1—H1A	0.9300	C14—C15	1.385 (7)
C2—C3	1.391 (6)	C14—C17	1.527 (6)
C2—H2A	0.9300	C15—C16	1.403 (5)
C3—C4	1.359 (6)	C15—H15A	0.9300
C3—C7	1.523 (5)	C16—C18	1.421 (7)
C4—C5	1.371 (5)	C17—H17A	0.9600
C4—H4A	0.9300	C17—H17B	0.9600
C5—C6	1.384 (5)	C17—H17C	0.9600
C5—H5A	0.9300	C18—H18A	0.9600
C6—C8	1.487 (4)	C18—H18B	0.9600
C7—H7A	0.9600	C18—H18C	0.9600
C7—H7B	0.9600		
			
O1—Cu1—O1^i^	180.00 (13)	N1—C9—N2	128.6 (3)
O1—Cu1—N1^i^	90.03 (11)	N1—C9—O3	115.1 (3)
O1^i^—Cu1—N1^i^	89.97 (11)	N2—C9—O3	116.3 (3)
O1—Cu1—N1	89.97 (11)	C9—O3—C10	118.9 (3)
O1^i^—Cu1—N1	90.03 (11)	O3—C10—H10A	109.5
N1^i^—Cu1—N1	180.0	O3—C10—H10B	109.5
C8—O1—Cu1	128.5 (2)	H10A—C10—H10B	109.5
C8—N2—C9	122.4 (3)	O3—C10—H10C	109.5
C8—N2—H2B	118.8	H10A—C10—H10C	109.5
C9—N2—H2B	118.8	H10B—C10—H10C	109.5
C9—N1—C11	116.6 (3)	C12—C11—C16	121.0 (3)
C9—N1—Cu1	122.7 (2)	C12—C11—N1	121.2 (4)
C11—N1—Cu1	120.7 (2)	C16—C11—N1	117.7 (3)
C6—C1—C2	120.7 (4)	C11—C12—C13	117.6 (5)
C6—C1—H1A	119.6	C11—C12—H12A	121.2
C2—C1—H1A	119.6	C13—C12—H12A	121.2
C1—C2—C3	121.4 (4)	C14—C13—C12	122.9 (5)
C1—C2—H2A	119.3	C14—C13—H13A	118.5
C3—C2—H2A	119.3	C12—C13—H13A	118.5
C4—C3—C2	117.6 (3)	C13—C14—C15	118.2 (4)
C4—C3—C7	121.9 (4)	C13—C14—C17	123.5 (5)
C2—C3—C7	120.6 (4)	C15—C14—C17	118.3 (6)
C3—C4—C5	121.2 (4)	C14—C15—C16	120.5 (5)
C3—C4—H4A	119.4	C14—C15—H15A	119.7
C5—C4—H4A	119.4	C16—C15—H15A	119.7
C4—C5—C6	121.5 (4)	C11—C16—C15	119.8 (4)
C4—C5—H5A	119.2	C11—C16—C18	121.0 (4)
C6—C5—H5A	119.2	C15—C16—C18	119.2 (5)
C1—C6—C5	117.5 (3)	C14—C17—H17A	109.5
C1—C6—C8	121.9 (3)	C14—C17—H17B	109.5
C5—C6—C8	120.4 (3)	H17A—C17—H17B	109.5
C3—C7—H7A	109.5	C14—C17—H17C	109.5
C3—C7—H7B	109.5	H17A—C17—H17C	109.5
H7A—C7—H7B	109.5	H17B—C17—H17C	109.5
C3—C7—H7C	109.5	C16—C18—H18A	109.5
H7A—C7—H7C	109.5	C16—C18—H18B	109.5
H7B—C7—H7C	109.5	H18A—C18—H18B	109.5
O1—C8—N2	127.0 (3)	C16—C18—H18C	109.5
O1—C8—C6	116.1 (3)	H18A—C18—H18C	109.5
N2—C8—C6	116.8 (3)	H18B—C18—H18C	109.5
			
O1^i^—Cu1—O1—C8	104 (100)	C5—C6—C8—N2	−179.4 (4)
N1^i^—Cu1—O1—C8	−178.2 (4)	C11—N1—C9—N2	−172.2 (4)
N1—Cu1—O1—C8	1.8 (4)	Cu1—N1—C9—N2	8.3 (6)
O1—Cu1—N1—C9	−7.7 (4)	C11—N1—C9—O3	4.2 (6)
O1^i^—Cu1—N1—C9	172.3 (4)	Cu1—N1—C9—O3	−175.3 (3)
N1^i^—Cu1—N1—C9	−128 (100)	C8—N2—C9—N1	−0.1 (7)
O1—Cu1—N1—C11	172.8 (3)	C8—N2—C9—O3	−176.4 (4)
O1^i^—Cu1—N1—C11	−7.2 (3)	N1—C9—O3—C10	−179.6 (4)
N1^i^—Cu1—N1—C11	53 (100)	N2—C9—O3—C10	−2.8 (6)
C6—C1—C2—C3	1.3 (8)	C9—N1—C11—C12	−82.1 (5)
C1—C2—C3—C4	−2.0 (8)	Cu1—N1—C11—C12	97.4 (4)
C1—C2—C3—C7	177.4 (5)	C9—N1—C11—C16	99.0 (4)
C2—C3—C4—C5	2.4 (8)	Cu1—N1—C11—C16	−81.5 (4)
C7—C3—C4—C5	−177.0 (5)	C16—C11—C12—C13	−1.0 (5)
C3—C4—C5—C6	−2.0 (8)	N1—C11—C12—C13	−179.9 (3)
C2—C1—C6—C5	−0.8 (7)	C11—C12—C13—C14	1.8 (6)
C2—C1—C6—C8	−175.5 (4)	C12—C13—C14—C15	−1.6 (6)
C4—C5—C6—C1	1.1 (7)	C12—C13—C14—C17	179.0 (4)
C4—C5—C6—C8	175.9 (4)	C13—C14—C15—C16	0.6 (6)
Cu1—O1—C8—N2	5.3 (7)	C17—C14—C15—C16	−179.9 (4)
Cu1—O1—C8—C6	−171.8 (3)	C12—C11—C16—C15	0.2 (5)
C9—N2—C8—O1	−7.5 (7)	N1—C11—C16—C15	179.1 (3)
C9—N2—C8—C6	169.6 (4)	C12—C11—C16—C18	178.9 (4)
C1—C6—C8—O1	172.6 (4)	N1—C11—C16—C18	−2.2 (6)
C5—C6—C8—O1	−2.0 (6)	C14—C15—C16—C11	0.1 (6)
C1—C6—C8—N2	−4.9 (6)	C14—C15—C16—C18	−178.8 (5)

Symmetry code: (i) −*x*, −*y*+1, −*z*.

### Hydrogen-bond geometry (Å, º)

**Table:**

*D*—H···*A*	*D*—H	H···*A*	*D*···*A*	*D*—H···*A*
C18—H18*A*···N1	0.96	2.27	2.783 (7)	112
